# Expanding the Clinical Phenotype Associated with the *NIN* Gene; Report of a Patient with Short Stature, Microcephaly and Hearing Loss

**DOI:** 10.34172/aim.33542

**Published:** 2025-05-01

**Authors:** Shima Zamanian Najafabadi, Zeinab Ghorbanoghli, Zhila Ghaderi, Fariba Afroozan, Ali Talea, Fatemeh Ahangari, Mina Makvand, Hossein Najmabadi, Ariana Kariminejad

**Affiliations:** ^1^Kariminejad-Najmabadi Pathology & Genetics Center, Tehran Iran; ^2^Metabolic Disorders Research Center, Endocrinology and Metabolism Molecular-Cellular Sciences Institute, Tehran University of Medical Sciences, Tehran Iran; ^3^Genetics Research Center, University of Social Welfare & Rehabilitation Sciences, Tehran, Iran

**Keywords:** Hearing loss, Microcephalic primordial dwarfism, MPD, NIN gene, Seckel syndrome

## Abstract

To date, there are very few reports regarding patients with bi-allelic variants in the *NIN* gene. There is one report of two sisters with severe short stature, microcephaly, and developmental delay with compound heterozygote missense variants in the *NIN* gene and one paper reporting a homozygote variant in the *NIN* gene with progressive, high-frequency sensorineural hearing loss in four siblings. The only other report is of four members of a consanguineous family with spondyloepimetaphyseal dysplasia with joint laxity-leptodactylic type (SEMDJL2) with a homozygous variant in the *NIN* gene. Given the scarcity of cases with *NIN* variants, the relationship between the phenotype and gene is provisional and our case broadens the phenotypic spectrum regarding the phenotype related to *NIN* gene variants. Here, we report a patient with a homozygous variant in exon 2 of the *NIN* gene defined as c.3407_3409del (p.Glu1136del). Clinical findings in our patient were characteristic of microcephalic primordial dwarfism (MPD) including microcephaly, prominent nose, intellectual disability and severe short stature. In addition, this patient had bilateral hearing loss, which was not reported in the patients with MPD and variant in the *NIN* gene before. We identified a novel p.Glu1136del variant in the *NIN* gene, predicted to disrupt critical centrosome-related pathways. WES was reanalyzed for other genes which are known for deafness and no variant was identified. A family history of deafness was not present in the pedigree. This is the first report of a patient with MPD and deafness associated with the *NIN* gene.

## Introduction

 Microcephalic Primordial Dwarfism (MPD) is characterized by severe pre- and postnatal growth failure associated with proportionate or disproportionate microcephaly associated with autosomal recessive inheritance.^1^ Syndromes included in MPD are Seckel syndrome (MIM 210600), osteodysplastic primordial dwarfism type 2 (MOPD2) (MIM# 210720), Meier-Gorlin Syndrome (MIM# 224690), Bloom syndrome (MIM# 210900), ligase IV deficiency (MIM# 606593) and XRCC4 deficiency (MIM# 616541).^1^ Seckel syndrome, one of the syndromes included in MPD, is characterized by intrauterine and postnatal growth retardation, proportionate microcephaly, intellectual disability and characteristic facial dysmorphism.^1^ Biallelic variants in the *ATR, RBBP8, CENPJ, CEP152, CEP63, DNA2, TRAIP, *and *NSMCE2* genes are associated with Seckel syndrome.^1^ Mendelian Inheritance in Man (MIM) has listed the *NIN* gene in association with Seckel syndrome 7 (MIM# 614851), however, mentioning that the relationship between the phenotype and the gene is provisional.

 Missense variants in the *NIN* gene have been previously reported in association with MPD (MIM614851), SEMDJL2 and one family with progressive high frequency hearing loss.^2-4^ Dauber et alreported two sisters with features of MPD with compound heterozygote variants in the *NIN* gene chr14:51224083T > C; c.3665A > G (pQ1222R)/chr14:51211022T > C; c.5126A > G p.N1709S.^2^ The sisters had severe prenatal and postnatal growth retardation, severe microcephaly, seizures and developmental delay. Growth hormone treatment was unresponsive. Bone age was delayed during childhood but reached chronological age at 14 years. Final height was in the range of -7 to -8 standard deviation (SD), weight was in the range of -3 to -4 SD and head circumference was in the range of -6 to -7 SD. There was shortening of limbs, more severe in lower limbs. They had clinodactyly of fifth fingers and Madelung deformity, lumbar scoliosis, and bilateral hip dysplasia. Hormonal study showed borderline central hypothyroidism. They had primary amenorrhea and Tanner 1 breast development. Facial dysmorphism included hypotelorism, slightly large nose and small ears. Dauber et alperformed functional studies showing *ninein* knockdown zebrafish had deformity of the neuroectoderm and organization of the midbrain-hindbrain boundary during early development of the larvae. They suggested that their patients should be classified as MPD and not Seckel or microcephalic osteodysplastic primordial dwarfism type II (MOPDII) on the basis of the relatively severe mental retardation which is usually mild to moderate in Seckel patients and mild to absent in MOPDII cases.

 Grosch et alidentified homozygous missense mutations in *NIN* (Ninein) and *POLE2* in four members of a Turkish consanguineous family with clinical and radiological features of SEMDJL2 which segregated in the family and was not present in 500 healthy control individuals.^3^ The proband had short stature (-5.2 SD), bilateral hip dysplasia, short arms and legs, laxity of small and large joints, genua valga, pes cavus, no intellectual disability, and mild facial dysmorphism. X-ray examination showed scoliotic spines, squared vertebral bodies with irregular end plates, bilateral hip dysplasia and dislocation, epiphyseal dysplasia and metaphyseal changes at the metaphyses of distal femur and proximal tibia. Hand X-rays showed slender (leptodactylic) metacarpals and phalanges. Based on the function of the *NIN* gene including chromosome congression during mitosis, centrosome maturation and centrosome architecture and its interaction with CEP110 which has an important role in remodeling microtubules and regulating ciliogenesis, they postulated that *NIN* is the more likely responsible gene, as genes encoding proteins affecting cilia formation and function have been associated with skeletal dysplasias.^5^

 Struass et alidentified a homozygous nonsense variant (c.4666C > T;p.Gln1556Ter) in four siblings with progressive high frequency hearing loss.^4^ No further information regarding the phenotype of the siblings was given. Based on functional studies, they postulated that variants in *NIN* could be responsible for hearing loss. They showed that *NIN* inhibits JAK2/STAT signaling, and this inhibition attenuates noise-induced hearing loss in mice. They also showed that Nin^-/-^ mice show isolated high frequency sensorineural hearing loss.

 Here, we report a patient with homozygous variant in the *NIN* gene with features overlapping with cases previously reported as MPD, SEMDJD2, and hearing loss.

## Subjects and Methods

 DNA was extracted from the patient’s peripheral blood using the standard salting-out method. Whole-exome sequencing was performed using the Twist Exome V2.0 capture system and sequenced on the NovaSeq 6000 platform according to the manufacturer’s protocols. According to CCDS Release 22, the human genome’s exons had a mean coverage depth of 200X, with 97.87% covered at 10X and 97.71% covered at 20X.

 The WES revealed a homozygous c.3407_3409del variant in the* NIN* gene located on chromosome 14. This variant results in an inframe deletion (p.Glu1136del) within the chromosome segregation ATPase domain of the NIN protein. Specific primers were designed for polymerase chain reaction (PCR) amplification (forward: CAACATCTCCTCTCTCAATGC, reverse: ATCACAGTCCGCACATAACAT), yielding a 572-bp product. The amplified PCR products were sequenced using an Applied Biosystems^TM^ 3130xl DNA Analyzer, and the sequences were compared to reference sequences using Codon Code Aligner (v8.0.1). Direct Sanger sequencing confirmed the homozygous state of the p.Glu1136del variant in the patient, while his parents were found to be heterozygous carriers.

 The identified variant, c.3407_3409del (p.Glu1136del), has not been reported in the Genome Aggregation Database (gnomAD), or the Iranian genomic population database (Iranome). *In-silico* prediction tools, including SIFT, PolyPhen-2, MutationTaster, MutationAssessor, FATHMM, and FATHMM-MKL, support the probable pathogenicity of this deletion, suggesting it is deleterious and disease-causing. According to the American College of Medical Genetics and Genomics (ACMG) guidelines, this variant is classified as of uncertain significance (PM2 + PP3), requiring further functional studies to conclusively determine its pathogenicity.

 This case highlights the importance of detailed genetic analysis in patients with complex congenital anomalies. The identification of a novel inframe deletion in the *NIN* gene (p.Glu1136del) underscores the need for further research to explore its role in the observed phenotype and its potential contribution to disease pathogenesis.

 Written informed consent for all studies, including permission to use photographs of the patients, was obtained from the subject’s parents.

 Structural Modeling and Analysis using UCSF Chimera: To assess the structural impact of the p.Glu1136del variant in the NIN protein, UCSF Chimera software (Version 1.10.2) was utilized for 3D structural visualization and analysis. The protein structure model was obtained from the EMBL-EBI database with PDB ID: AF-Q8N4C6-F1-v4. Structural analysis focused on changes in the root mean square deviation (RMSD), hydrogen-bond network, hydrophobicity, and B-factor surrounding the mutation site. This allowed for evaluation of localized structural perturbations that may affect protein-protein interactions, particularly with CEP170.

 Pathway Analysis: BioReactome was utilized to explore the molecular pathways potentially affected by the *NIN* p.Glu1136del variant. Through this tool, we identified significant pathways involving centrosomal function, CRMPs in Semaphorin signaling, HSF1-mediated heat shock response, and ubiquitin-dependent degradation of Cyclin D (Table 1).

**Table 1:** Significant Pathways Identified from BioReactome Analysis of the *NIN* Gene (p.Glu1136del Variant)

**Pathway Name**	**Entities Found Ratio**	**Entities ** ***P*** **-Value**	**FDR**	**Reactions Found Ratio**	**Reactions** ***P *** **Value**
CRMPs in Sema3A signaling	1/73	0.004	0.011	1/5	0.00033
GSK3B and BTRC: CUL1-mediated-degradation of NFE2L2	1/95	0.005	0.014	1/4	0.000264
Ubiquitin-dependent degradation of Cyclin D	1 / 115	0.006	0.017	1/5	0.00033
AXIN missense mutants destabilize the destruction complex	1 / 142	0.007	0.021	1/1	6.59E-05
Signaling by AXIN mutants	1 / 142	0.007	0.021	1/2	0.000132
APC truncation mutants have impaired AXIN binding	1 / 165	0.008	0.024	1/1	6.59E-05
Signaling by APC mutants	1 / 165	0.008	0.024	1/2	0.000132
Truncations of AMER1 destabilize the destruction complex	1 / 182	0.009	0.027	1/1	6.59E-05
Maturation of nucleoprotein	1 / 178	0.009	0.026	1/9	0.000594
Signaling by AMER1 mutants	1 / 182	0.009	0.027	1/2	0.000132
Degradation of GLI2 by the proteasome	1 / 193	0.01	0.029	1/5	0.00033
GLI3 is processed to GLI3R by the proteasome	1 / 196	0.01	0.029	1/5	0.00033
Kinesins	1 / 200	0.01	0.03	1/14	0.000923
Signaling by WNT in cancer	1 / 228	0.011	0.034	3/16	0.001
Semaphorin interactions	1 / 218	0.011	0.032	1/41	0.003
Disassembly of the destruction complex and recruitment of AXIN to the membrane	1 / 270	0.013	0.04	1/7	0.000462
Regulation of HSF1-mediated heat shock response	2 / 264	0.013	0.000513	4/14	0.000923
Regulation of RUNX2 expression and activity	1 / 298	0.015	0.044	1/29	0.002
Cellular response to heat stress	2 / 351	0.017	0.000904	4/29	0.002
Hedgehog 'off' state	1 / 360	0.018	0.053	2/32	0.002
Activation of AMPK downstream of NMDARs	1 / 373	0.019	0.055	2/3	0.000198
TCF dependent signaling in response to WNT	2 / 1,747	0.087	0.021	3/71	0.005
Signaling by WNT	2 / 2,209	0.11	0.034	4/157	0.01

FDR, False discovery rate.

 VarSome was used for further functional interpretation of the p.Glu1136del variant. This tool provided additional insights into the variant’s pathogenicity by integrating data from multiple *in-silico* prediction algorithms, supporting the hypothesis that this variant may disrupt critical protein interactions, particularly within the centrosome.

## Clinical Description

**Figure 1:**
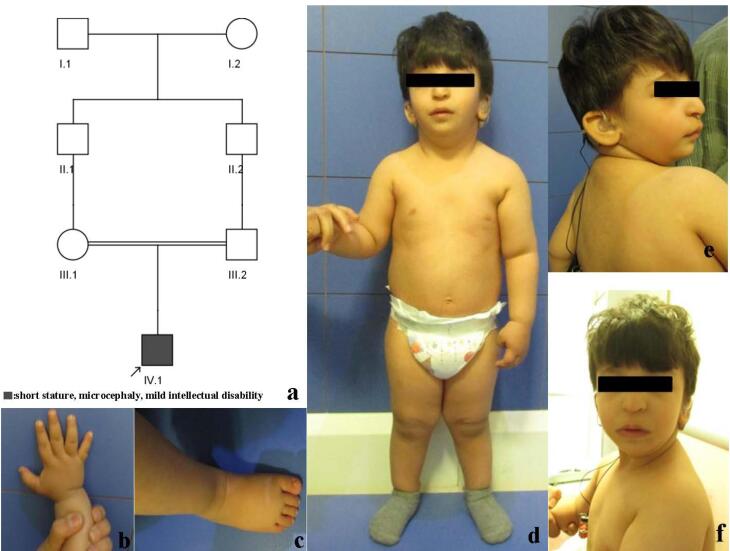
**a)** Pedigree, **b and c)** Hand and Foot. Note short middle phalanx of fifth finger, **d)** Full length frontal view, lateral and frontal view of face. Note short stature

 The proband is the first and only child of apparently healthy first-cousin parents. Ultrasound examination at 28 weeks of pregnancy reported intra-uterine growth retardation (IUGR). Delivery was by cesarean section at 38 weeks. The Apgar score was 9. Birth measurements were; weight 2260 grams (3^rd^ centile, -2.07 SD, length 48 cm ( < 25^th^ centile, -1.05SD) and head circumference 31 cm < 2^nd^ centile, -3 SD). Microcephaly, hearing loss and poor feeding were noted days after birth. He was hospitalized at 40 days after birth with fever and failure to thrive. Milestones were delayed, held head at 3‒4 months, rolled at 1 year, crawled at 15 months, stood at 1 year and walked at 19 months. He could say his first word at 18 months. Examination at 2 years and 7 months showed weight 11.5 kg (8‒9^th^ centile, -1.5 SD), length 79 cm ( < 3^rd^ centile,-3.5 SD) and head circumference 42.5 cm (-4 SD). He was alert and made good contact with others. He could say about 20‒30 words. There was mild facial dysmorphism with downslanting and short palpebral fissures, large nose and prominent tip, micrognathia and low-set and posteriorly rotated ears. There were no apparent skeletal anomalies. Short fifth middle phalanx was noted. Phallus was small and testes were undescended. Surgery for correction of bilateral cryptorchidism was performed at 2 years and 4 months (Figure 1).

 Paraclinical examination: Brain CT was performed at 2.5 months but was not very helpful since fontanels were closed. Ultrasound examination of the pelvis, kidneys, bladder, testes and inguinal canal was performed at 1 month and 3 days. The hip was normal and did not show dysplasia. The kidneys were normal in size and shape.

 Brain MRI at 5 months was normal. Electroencephalogram was normal. Echocardiography at 5 months did not reveal any abnormality other than a patent foramen ovale. Auditory brainstem response (ABR) performed at at one year and seven months showed moderate hearing loss in the right ear and severe to profound hearing loss in the left ear.

 Hormonal tests showed normal T3, T4, TSH, FSH, LH, Testosterone, Estradiol, Androstenedione, dihydrotestosterone, Insulin-Like Growth Facor 1, Growth Hormone (Fasting), 17 Hydroxyprogesterone levels. Cortisol (8:00 AM), ACTH and Plasma direct (Active) Renin were elevated [17.2 microgram/dL (normal range 4.0‒16.5 microgram/dL), 137 pg/mL (Normal range: up to 46), and 101.5 microIU/mL (4.2‒60), respectively] and he was treated with hydrocortisone. ACTH and Renin reached the normal range after treatment.

## Discussion

 In this study, we report a patient with short stature, microcephaly and hearing loss with a novel homozygous variant in the *NIN* gene. This gene has been reported in association with three different phenotypes, MPD, SEMDJL2 and progressive sensorineural hearing loss.^2-4^

 Dauber et al reported two sisters with MPD carrying compound heterozygous NIN variants. They exhibited severe growth retardation, microcephaly, developmental delay, seizures, and skeletal abnormalities.^2^ Functional studies in zebrafish showed neuroectodermal defects, leading the authors to classify the condition as MPD rather than Seckel syndrome or MOPDII.

 Grosch et al identified *NIN* and *POLE2* mutations in a consanguineous Turkish family with SEMDJL2.^3^ The proband had short stature, joint laxity, limb abnormalities, and hip dysplasia but no intellectual disability. Given *NIN*’s role in centrosome maturation, the authors suggested it as the likely causative gene.

 Strauss et al identified a nonsense *NIN* variant in four siblings with progressive high-frequency hearing loss.^4^ Functional studies indicated that NIN inhibits JAK2/STAT signaling, which may protect against noise-induced hearing loss.

 Here, we report a patient with homozygous variant in the *NIN* gene with features overlapping with cases previously reported, highlighting their similarities and differences.

 Short stature is a feature common to all reported cases, being more severe in the two siblings reported as MPD and SEMDJL2 in comparison to our patient with -6‒7 SD for the MPD patients aand -4.7 SD for the SEMDJL2 patient while ours had a SD of -3. There was no information on the clinical features of the patient with hearing loss so unfortunately, we cannot compare it to the other patients. Microcephaly was most severe in the MPD patients with a -6.7 SD while ours was in the range of -3.7SD and the SEMDJL2 patient was on the low normal percentile and a -1.7 SD. IUGR was present in all patients with birth weights less than the 3^rd^ centile. Mild facial dysmorphism including large nose was noted in these patients.

 Skeletal abnormalities were present in the patients with MPD and SEMDJL2, being more severe in the patient with SEMDJL2. Shortening of limbs, lumbar scoliosis and bilateral hip dysplasia were noted in both patients. Our patient did not show any obvious signs of skeletal involvement and did not consent to radiological examination. Intellectual disability was seen in the two siblings with MPD and our patient, being more severe in the two MPD siblings. Hormonal imbalance was also a feature common to the MPD siblings and our patient. The two MPD siblings showed primary amenorrhea, absent breast development and central hypothyroidism, while our patient had elevated cortisol, increased ACTH and renin.

 Hearing loss was present in our patient and in one family with four affected siblings with progressive sensorineural hearing loss. The clinical features of all patients with variants in the *NIN* gene are summarized in Table 2. In summary, our patient has features overlapping with all previously reported cases and it seems that clinical features present in patients with biallelic variants in the *NIN* gene show a spectrum of abnormalities and not one specific phenotype.

**Table 2:** Clinical Features of all Patients with Variants in the *NIN* Gene

	**Dauber et al, 2012**		**Grosch et al, 2013**	**Strauss et al, 2017**	**Present Case**
	**Case 1**	**Case 2**	**Case 1**	**Case 3‒6**	**Case 7**
Ethnicity	Indian	Indian	Turkish	N/A	Iranian
Age at last examination	22 years	18 years	35 years		2 years and 7 months
IUGR	+	+	+	NA	+
Gender	Female	Female	Female	2Male/2Female	Male
Weight at birth (g/percentile)	1590g (-3.11 SD)	2017g (-2.33 SD)	Small for gestational age	NA	2.260g (-2.07 SD)
Length at birth (cm/SDS)	Unknown	38.1 (-6 SD)	45 cm (-2.37 SD)	NA	48 (-1.05 SD)
Head circumference at birth (cm)	NA	NA	NA	NA	31 (-3 SD)
Weight at examination (kg)	35.5 (-2.95 SD)	38 (-2.2 SD)	34 (-3.1 SD)	NA	11.5 (-1.48 SD)
Length at examination (cm)	115 ( < -6.79 SD)	106.7 (-7.97 SD)	129 (-4.79 SD)	NA	79 (-3.09 SD)
Head circumference at examination (cm/SDS)	44.8cm (-6.8 SD) at 22 years	45 (-6.7 SD)	52 (-1.71 SD	NA	42.5 (-3.71 SD)
Eyes	NA	NA	NA	NA	Downslantig and short palpebral fissures
Vision	Normal	Normal	Normal	NA	
Nose	Slightly large nose	Slightly large nose	Short, flat nasal bridge	NA	Large nose, Prominent nasal nose
Ears	Small ears	Small ears	NA	NA	Low-set and posteriorly rotated
Hearing	Normal	Normal	Normal	Progressive high frequency hearing loss	Moderate hearing loss in right ear and severe to profound hearing loss in left ear
Jaw	NA	NA	NA	NA	-
Teeth	NA	NA	Carious	NA	-
Limbs	Short limbs, with legs affected more than arms		Epiphyseal and metaphyseal dysplasia of long bones	NA	-
Puberty	Absent breast development, primary amenorrhea	Absent breast development, primary amenorrhea	NA	NA	NA
Radiography of hand			Slender metacarpals and phalanges (leptodactyly)	NA	NA
Finger			Slender		NA
Spine	Lumbar scoliosis, mild	Lumbar scoliosis, mild	Scoliotic spine, squared vertebral bodies and irregular end plates	NA	NA
Hip	Bilateral hip dysplasia	Bilateral hip dysplasia	Bilateral hip dysplasia and dislocation	NA	Normal
Extremities	Madelung deformity	Madelung deformity	Poor modeling and fine vertical striations at the metaphyses of distal femur and proximal tibia	NA	NA
Intellectual disability	Preschool level (at age 22yr)	Preschool level (at age 18yr)	—	NA	+
Seizure presence	Yes	Yes	NA	NA	NO
Age of onset	5 months	18 months	NA	NA	—
Hormonal study	Central hypothyroidism	Central hypothyroidism, poor response to growth hormone	NA	NA	Increase in Renin and ACTH level (returned to normal with hydrocortisone treatment)
Variant	c.3665A > G (p.Gln1222Arg)/ c.5126A > G (p.Asn1709Ser)		c.6244A > G (p.Asn2082Asp)	c.4666 C > T, p.(Gln1556*)	c.3407_3409del (p.Glu1136del)
Zygosity	Compound heterozygotes		homozygote	Homozygote	Homozygote
Classification	VUS		VUS	Likely Pathogenic	VUS
Other	Subglottic stenosis				

NA, not available.

 The phenotype/s associated with the *NIN* gene are not clearly defined yet. Further cases need to be reported to determine if it would be more appropriate to define one phenotype with a spectrum of abnormalies including pre- and postnatal growth retardation, microcephaly, intellectual disability, skeletal anomalies, hormonal imbalance and hearing loss, or define more than one phenotype that can be associated with the *NIN* gene.

**Figure 2:**
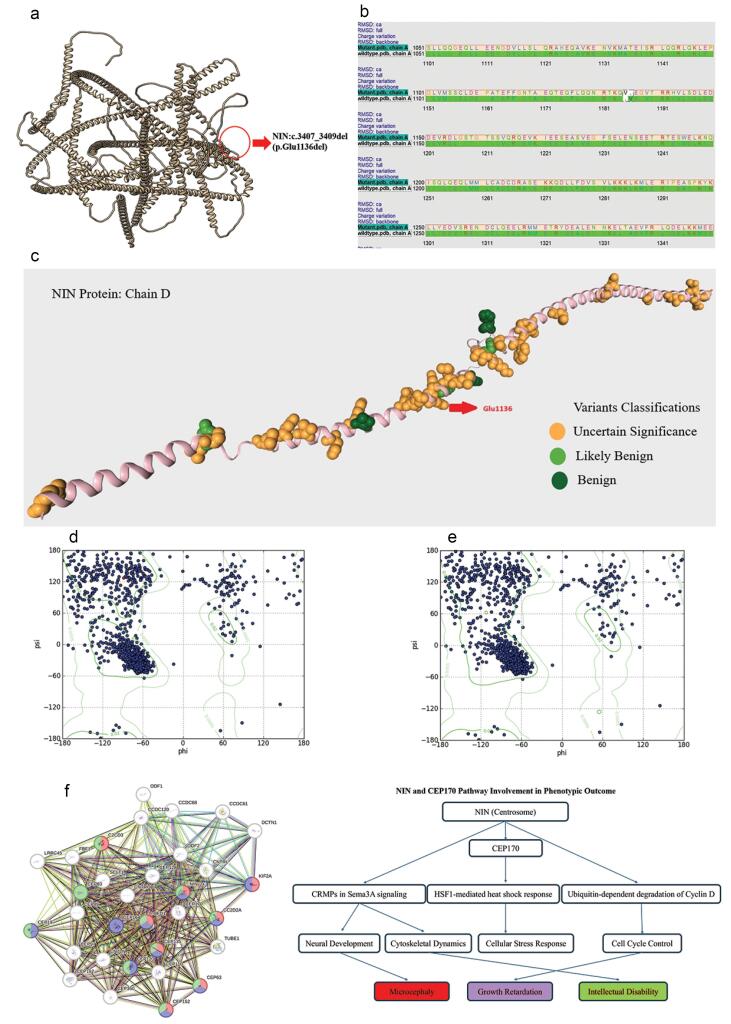
**a)** Superposition of Wild-Type and Mutant (p.Glu1136del) NIN Proteins: The superposition of wild-type and p.Glu1136del mutant NIN proteins performed using MatchMaker shows no significant differences in hydrogen bonding or B-factor values. The only notable structural change is a decrease in hydrophobicity near the deletion site, potentially affecting local stability or interactions. **b)** Sequence Alignment and Structural Comparison between Wild-Type and Mutant (p.Glu1136del) NIN Protein: The sequence alignment of the wild-type NIN protein (chain A) and its mutant form (p.Glu1136del) highlights the deletion at position 1136. The gap in the mutant sequence (Mutant.pdb, chain A) corresponds to the loss of the Glu1136 residue. RMSD values for the C-alpha atoms, full structure, and backbone are provided to assess structural deviations between the wild-type and mutant proteins. Charge variations are highlighted in color across the sequence, with no significant changes observed despite the deletion. The overall alignment shows conservation in sequence, but local structural effects from the mutation may influence protein function. **c)** VARSOM Annotation of NIN Protein (Chain D) Highlighting Variant Classifications: The NIN protein (Chain D) is annotated with variants classified by VARSOM. The variant at position Glu1136 is marked by a red arrow, with nearby variants shown in orange, indicating they are of Uncertain Significance (VUS). Variants classified as likely benign are shown in light green, and benign variants are represented in dark green. This annotation demonstrates that most variants surrounding the Glu1136 position are classified as VUS, supporting the need for further functional studies on the p.Glu1136del mutation. **d)** Ramachandran Plot of Wild-Type NIN Protein: The Ramachandran plot for the wild-type NIN protein shows a dense clustering of residues in the allowed regions, primarily within the alpha-helix region (-60°, -40°) and the beta-sheet region (-120°, + 120°). The overall conformation is stable with minimal outliers in disallowed regions, reflecting proper protein folding. **e)** Ramachandran Plot of Mutant NIN Protein (p.Glu1136del): The Ramachandran plot for the NIN p.Glu1136del mutant reveals a noticeable shift in the distribution of phi (ϕ) and psi (ψ) angles. While the beta-sheet region remains largely intact, there is a reduced density in the alpha-helical region, indicating potential instability in this structural motif. Additionally, more residues are observed in disallowed regions, suggesting local structural disruptions caused by the deletion mutation. **f)** Pathway Enrichment Analysis of NIN and CEP170 using BioReactome and STRING DB. This diagram illustrates the pathway enrichment analysis for NIN and CEP170 proteins. The network on the left was generated using STRING DB, showing protein-protein interactions, while the pathway flowchart on the right highlights key biological processes associated with these proteins. The colors represent associations with specific human phenotypes based on the Human Phenotype Ontology (HPO). (Red nodes indicate genes involved in microcephaly; Purple nodes are linked to growth retardation; Green nodes correspond to genes associated with intellectual disability) The pathways involving NIN and CEP170 include roles in cytoskeletal dynamics, cell cycle control, neural development, and cellular stress response, ultimately leading to phenotypic outcomes such as microcephaly, growth retardation, and intellectual disability

 The alignment between the wild-type and mutant (p.Glu1136del) NIN proteins highlights a deletion at position 1136, where the wild-type protein possesses a glutamic acid residue (Glu), which is absent in the mutant. Despite this deletion, the surrounding sequences remain conserved, and no significant charge variations are observed across the sequence. The structural analysis of the NIN protein revealed no significant alterations in the hydrogen-bond network following the p.Glu1136del variant (Figure 2a). Despite the absence of large-scale structural perturbations, a notable decrease in the hydrophobicity score was observed near the mutation. This suggests localized destabilization, particularly in regions involved in protein-protein interactions. Furthermore, the B-factor remained unchanged at 20.36, implying that the mutation does not significantly affect atomic displacement or flexibility in the region. RMSD calculations, focusing on the C-alpha atoms, full structure, and backbone, suggest that the overall structural impact of the mutation is minimal in most regions, indicating that the protein retains its general fold. However, local conformational changes around the site of deletion may still affect the mutant’s stability or function, as suggested by changes in secondary structure, particularly in the alpha-helical regions (Figure 2b). This comparison demonstrates that while the deletion of Glu1136 does not drastically alter the global structure, it may cause localized instability or flexibility that could impact the protein’s functional dynamics.

 The deleted residue, Glu1136, is located within a critical region (K802-M1505) that plays an important role in interacting with CEP170 (9). CEP170 is crucial for centrosome function, and the disruption of this interaction could potentially impair centrosomal architecture and function.^6^ This may align with the phenotypic consequences observed in the patient, such as microcephaly and primordial dwarfism. Notably, previous variants near Glu1136 have been classified as variants of uncertain significance (VUS)^7^ (Figure 2c), further supporting the possibility that this mutation has a functional impact, despite the absence of large-scale structural changes.

 Although the p.Glu1136del variant does not drastically alter the overall structural integrity of the NIN protein, its location in a region crucial for CEP170 interaction and the observed hydrophobicity changes suggest that the mutation could disrupt essential molecular interactions required for centrosome maturation and function. This may contribute to the patient’s clinical presentation. However, further functional studies are necessary to confirm these findings and fully understand the biological impact.

 Ramachandran plots were generated for both the wild-type (WT) NIN protein and its mutant form (p.Glu1136del) to assess the impact of the deletion on the overall structural stability and secondary structure formation.

 The Ramachandran plot of the wild-type NIN protein (Figure 2d) exhibited a well-defined distribution of phi (ϕ) and psi (ψ) angles, with most residues clustering in the expected allowed regions. A strong presence in the alpha-helical region (-60°, -40°) and beta-sheet region (-120°, + 120°) indicates that the wild-type protein adopts stable secondary structures with limited outliers in disallowed regions.

 In contrast, the p.Glu1136del mutant (Figure 2e) displayed notable changes in its Ramachandran plot, suggesting a perturbation in its structural conformation. While the beta-sheet region remained largely unaffected, a significant reduction in the density of residues within the alpha-helical region was observed, indicating a potential destabilization or partial unfolding of alpha-helical domains. Moreover, the mutant plot showed an increased number of residues occupying disallowed regions, which suggests the introduction of unfavorable conformations and possible local destabilization due to the deletion of Glu1136.

 These findings suggest that the p.Glu1136del mutation leads to a loss of stability in the alpha-helical regions while largely preserving the beta-sheet architecture, potentially contributing to altered protein function or folding dynamics.

 In addition to these structural findings, several predicted pathways have been identified through bioinformatic analysis, which may offer additional insights into the possible mechanisms underlying the patient’s phenotype (Figure 2f).^8^ These pathways, though speculative, suggest potential cellular processes that could be disrupted by the mutation, particularly in relation to NIN-CEP170 interactions and the clinical manifestations of MPD.

 One of the top predicted pathways is the regulation of HSF1-mediated heat shock response (*P* value = 0.013) and cellular response to heat stress (*P* value = 0.017). Both pathways involve heat shock proteins (HSPs), which play a key role in maintaining cellular homeostasis during stress. Since NIN is involved in regulating cellular architecture and mitotic processes, it may be particularly susceptible to stress conditions, especially if HSPs that interact with centrosomal proteins are affected.^9^ While no significant RMSD changes were observed in the structural analysis, the localized hydrophobicity changes and the involvement of stress-response pathways suggest that the NIN-CEP170 complex may be less able to cope with cellular stress, potentially contributing to developmental defects such as microcephaly and growth retardation.

 Another significant pathway, CRMPs in Sema3A signaling (*P* value = 0.004), highlights a possible link between centrosomal dynamics and neural development. Collapsin response mediator proteins (CRMPs) are integral to semaphorin signaling, which is critical for neuronal growth and axonal guidance.^10^ Disruption of CRMPs can lead to altered cytoskeletal dynamics, which may impair normal neuronal development. Given NIN’s role in centrosome maturation and CEP170’s involvement in microtubule organization,^11^ aberrant interactions influenced by this pathway could contribute to the microcephaly and intellectual disability observed in the patient.^12^ However, further experimental validation is required to establish a direct connection between these proteins and CRMP-mediated signaling.

 The ubiquitin-dependent degradation of Cyclin D (*P* value = 0.006) is another pathway of interest. Cyclin D is a key regulator of the cell cycle, and its dysregulation can lead to abnormal cell proliferation.^13^ Centrosomal proteins such as NIN and CEP170 influence mitotic progression, and it is plausible that the p.Glu1136del variant perturbs cell cycle dynamics, particularly during the early development of neural progenitor cells.^14^ This disruption could manifest as microcephaly and growth failure, which are prominent features of the patient’s condition. Although speculative, the link between centrosomal dysfunction and impaired cell cycle regulation provides a plausible explanation for the severe prenatal and postnatal growth retardation observed.

 Lastly, the semaphorin interactions pathway (*P* value = 0.011) further supports the role of sema3A signaling in the patient’s phenotype. Semaphorins and their associated proteins, such as CRMPs, are vital for both neural development and cytoskeletal arrangement—processes that rely heavily on functional centrosomes.^15^ Disruption in Semaphorin signaling could exacerbate cytoskeletal abnormalities, contributing to the skeletal anomalies and intellectual disability observed in the patient.^16^ Since NIN is essential for maintaining centrosomal architecture, even minor changes in its function could have downstream effects on these developmental processes.

## Conclusion

 In summary, while the pathways identified through bioinformatic analysis are speculative and require further experimental validation, they provide a potential framework for understanding the broader effects of the p.Glu1136del variant in *NIN*. The involvement of pathways related to the heat shock response, CRMP-mediated signaling, and cell cycle regulation suggests complex interactions between centrosomal proteins and cellular stress mechanisms. These pathways could collectively contribute to the observed phenotype of short stature, microcephaly, intellectual disability, growth retardation and hearing loss. Future studies focusing on these pathways may help to elucidate the precise molecular mechanisms underlying this rare genetic condition.

 With this report, we expand the phenotype related to bi-allelic variants in the *NIN* gene. However, more cases should be reported to determine the phenotype or phenotypes related to this gene.
